# Circulating biomarkers and mortality in atrial fibrillation: the REasons for Geographic And Racial Differences in Stroke study

**DOI:** 10.1093/ehjopen/oeag022

**Published:** 2026-02-17

**Authors:** Erin M Hald, Katherine Wilkinson, Samuel A P Short, Suzanne E Judd, Virginia J Howard, Emily B Levitan, Elsayed Z Soliman, Mary Cushman

**Affiliations:** Department of Medicine, Larner College of Medicine at the University of Vermont, 89 Beaumont Ave, Burlington, VT 05405, USA; Thrombosis Research Group (TREC), Department of Clinical Medicine, UiT-The Arctic University of Norway, 9037 Tromsø, Norway; Division of Internal Medicine, University Hospital of North Norway, 9038 Tromsø, Norway; Department of Medicine, Larner College of Medicine at the University of Vermont, 89 Beaumont Ave, Burlington, VT 05405, USA; Department of Medicine, Larner College of Medicine at the University of Vermont, 89 Beaumont Ave, Burlington, VT 05405, USA; Department of Biostatistics, University of Alabama at Birmingham, 1665 University Boulevard, Room 327, Birmingham, AL 35294, USA; Department of Epidemiology, University of Alabama at Birmingham, 1720 2nd Ave S, Birmingham, AL 35294-0022, USA; Department of Epidemiology, University of Alabama at Birmingham, 1720 2nd Ave S, Birmingham, AL 35294-0022, USA; Epidemiological Cardiology Research Center, Department of Internal Medicine, Section on Cardiovascular Medicine, Wake Forest School of Medicine, 525 Vine St, Winston-Salem, NC 27101, USA; Department of Medicine, Larner College of Medicine at the University of Vermont, 89 Beaumont Ave, Burlington, VT 05405, USA

**Keywords:** Atrial fibrillation, Mortality, Biomarkers, Cohort study

## Abstract

**Aims:**

Examination of biomarkers associated with mortality among people with atrial fibrillation (AF) may help identify possible preventive interventions in this high-risk population. We aimed to study associations of circulating biomarkers with all-cause and cause-specific mortality in persons with AF in the US national biracial REasons for Geographic And Racial Differences in Stroke (REGARDS) cohort.

**Methods and results:**

REGARDS enrolled 30 239 Black and White adults aged ≥45 in 2003–07. Candidate biomarkers were measured in all participants with baseline AF and no prior stroke (*n* = 2260) and deaths identified through 31 December 2019. We calculated hazard ratios (HRs) with 95% confidence intervals (CIs) for all-cause, cardiovascular-, and cancer-related mortality by biomarker levels. The mean baseline age was 67.5 years. Participants were 53.5% female, 35.7% identified as Black, and 21.3% were taking an anticoagulant. Over 10.3 years, 1151 participants died (38.7% of cardiovascular disease, 16.1% of cancer). In multivariable-adjusted analyses, all analysed biomarkers except lipoprotein(a) were associated with all-cause mortality (HR, 95% CI for fourth vs. first quartile): N-terminal pro B-type natriuretic peptide (4.85; 3.70–6.36), galectin-3 (2.03; 1.65–2.51), growth differentiation factor 15 (3.98; 3.00–5.29), cystatin C (2.81; 2.21–3.58), interleukin-6 (2.61; 2.08–3.26), D-dimer (1.74; 1.40–2.15), γ-glutamyltransferase (1.46; 1.21–1.76), and factor VIII antigen (2.03; 1.65–2.50). Most biomarker associations were stronger for cardiovascular than cancer mortality and did not differ by race.

**Conclusion:**

Several biomarkers were associated with all-cause and cardiovascular mortality in AF, suggesting multiple domains of clinical relevance that support interventions to reduce mortality.

What’s New?

- Patients with AF are at elevated risk for mortality, but the prognostic value of specific circulating biomarkers in this population is not fully established.
- In a large, biracial US cohort, several biomarkers reflecting cardiac stress, inflammation, renal dysfunction, and coagulation were independently associated with all-cause and cardiovascular mortality in individuals with AF.
- Our findings suggest that incorporating biomarker assessment in individuals with AF may facilitate more precise interventions aimed at reducing mortality.

## Introduction

Atrial fibrillation (AF) is the most frequent cardiac arrhythmia, affecting close to 40 million people worldwide.^1^ Despite advances in treatment, AF remains a major public health burden, contributing substantially to morbidity and mortality. Over the last decades, the AF-related death rate has increased more than the AF incidence rate globally,^2^ and AF contributes to 230 000 deaths annually in the USA.^3^

Contemporary treatment strategies for AF have largely focused on optimizing anticoagulant therapy, as it effectively reduces stroke risk and stroke-related mortality. In a recent meta-analysis, stroke-related deaths nevertheless only accounted for 6% of total deaths among persons with AF, while the majority died of heart disease.^4^ Death was the most frequent adverse outcome during follow-up in clinical AF trials and registries, occurring at a three-fold higher rate than stroke.^5,6^ The high mortality burden in AF warrants further efforts to identify factors associated with death in this patient group.

Emerging evidence has implicated circulating biomarkers as associated with all-cause and cardiovascular (CVD) mortality in AF. Markers of cardiac dysfunction and fibrosis, oxidative stress and inflammation, renal dysfunction, and coagulation activation have all been associated with AF mortality,^7–11^ and information on such biomarkers may improve the pathophysiological understanding of AF and aid in risk assessment. Most data on these biomarkers and AF mortality are derived from the clinical trials of anticoagulants^8,11^ and may not accurately reflect the general AF population. A recent review article found that the pooled participation of African Americans in trials reporting participant-level data was only 2%,^12^ while Black or African American people constitute 14% of the US population.

Despite a reported lower AF incidence in Black individuals compared to White individuals, previous studies have demonstrated higher rates of adverse outcomes, including overall and cardiovascular mortality, in Black individuals with AF.^13–15^ In the Atherosclerosis Risk in Communities (ARIC) cohort, Black individuals with AF had a significantly higher risk of all-cause mortality than White individuals.^15^ In another large study of Medicare-enrolled older patients with AF, the risk of death was 1.5-fold greater for Black compared to White individuals, a difference explained by comorbidities.^16^ In the biracial REasons for Geographic And Racial Differences in Stroke (REGARDS) cohort, participants with AF had a 32% greater risk of mortality compared to those without AF, with similar risks in Black and White participants.^17^ To the best of our knowledge, the impact of specific biomarkers on mortality in AF by race group has not been reported previously.

For the present research, nine blood biomarkers were selected based on reported associations with adverse outcomes in AF^8,10,18–20^ and measured in participants with prevalent AF in the REGARDS cohort. We aimed to investigate the associations between these biomarkers and all-cause and cause-specific (cardiovascular and cancer) mortality.

## Methods

### Study population and design

The REGARDS study is a prospective, population-based cohort study designed to investigate regional and Black–White disparities in stroke in the USA. In this report, we consider race as a sociopolitical variable, not a biological variable. A detailed description of the REGARDS study has been published previously.^21^ Briefly, 30 239 participants aged ≥45 years were recruited between 2003 and 2007, with intentional oversampling of Black persons (44%) and persons residing in the southeastern ‘Stroke Belt’ (56%), a region of high stroke mortality encompassing Alabama, Arkansas, Georgia, Louisiana, Mississippi, North Carolina, South Carolina, and Tennessee. Baseline data on demographics and medical history were obtained by telephone interview, and the participants were subsequently visited in-home by trained personnel who measured anthropometrics, collected fasting blood samples, and performed an electrocardiogram (ECG) and a medication inventory. Participants are followed by telephone contact every 6 months, and in 2013–16, a second extended phone interview and in-home examination were conducted. The study was approved by institutional review boards at each participating site, and all participants provided written informed consent.

### Inclusion criteria for the present analysis

**Figure 1:**
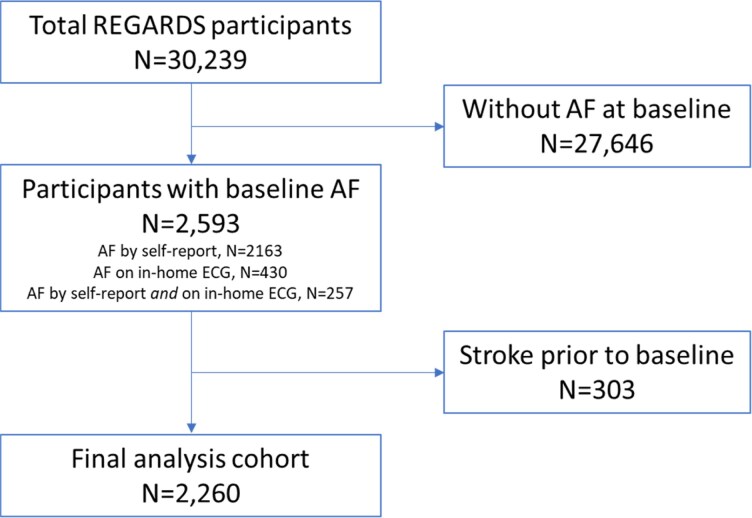
Assembly of the study cohort from the REGARDS (REasons for Geographic And Racial Differences in Stroke) participants.

In the present study, all participants who had AF and no prior stroke at the first REGARDS in-home visit were included (*Figure 1*). Atrial fibrillation was identified by either self-report or the study ECG. All ECGs were centrally interpreted by trained electrocardiographers blinded to clinical data. Self-reported AF was defined as an affirmative response to the question ‘Has a physician or a health professional ever told you that you had atrial fibrillation?’ A previous REGARDS study found that self-reported and ECG-verified AF were similarly predictive of stroke.^22^ For this study, we did not differentiate between paroxysmal, persistent, and permanent AF.

### Biomarker measurements

Blood samples from participants were drawn at the baseline in-home visit and centrifuged near participants’ homes, shipped overnight on ice to the University of Vermont central laboratory, and then re-centrifuged and stored at −80°C.^23^ Biomarkers were selected based on previously reported associations with adverse outcomes in AF and pilot data from this cohort related to stroke risk.^19^ We studied NT-proBNP (N-terminal pro-B-type natriuretic peptide), galectin-3, GDF-15 (growth differentiation factor 15), IL-6 (interleukin-6), coagulation factor VIII, D-dimer, cystatin C, GGT (γ glutamyltransferase), and lipoprotein(a). Details on the measurement methods and assay specifications for the biomarkers studied are provided in the Supplementary material.

### Outcome assessment

Information on all-cause mortality and cause-specific (cardiovascular and cancer) mortality was obtained through biannual follow-up by telephone of all study participants, linkage with the Social Security Death Index and the National Death Index, as well as medical records and death information from the participants’ proxies.^21^ Time to death was determined based on date of death from death certificate, the Social Security Death Index, or/and the National Death Index. The cause of death was defined as the one disease or injury that initiated the events resulting in death and centrally adjudicated by trained clinicians as previously described in detail.^24^ Cardiovascular death was defined as death from coronary heart disease, stroke, heart failure, sudden death, vascular pathology, and other cardiovascular causes (e.g. aortic dissection), and cancer death was defined as death from any type of cancer; for the present analysis, we did not look at specific cancer types.

### Covariates

Information on age, race, sex, income, education, geographic region of residence, and smoking was obtained by self-report. Anthropometric measures and information on medication use were obtained at the in-home visit, and BMI was calculated as weight (kg)/height (m^2^). Blood pressure was measured using a standard aneroid sphygmomanometer after 5 min of seated rest. Two recordings were taken, and the mean of the two measurements was used for analysis. Hypertension was defined as systolic blood pressure ≥ 140 mmHg, diastolic blood pressure ≥ 90 mmHg, and/or use of antihypertensive medications. Diabetes mellitus was defined as fasting blood glucose > 126 mg/dL, non-fasting blood glucose > 200 mg/dL, and/or use of antidiabetic agents. Prior coronary artery disease was defined as self-reported history of prior myocardial infarction, coronary artery bypass surgery, coronary angioplasty or stenting, and/or ECG evidence of myocardial infarction at baseline. Persons were classified as having dyslipidaemia if total cholesterol or LDL cholesterol ≥ 240 and 160 mg/dL, respectively, HDL cholesterol ≤ 40 mg/dL, or self-reported use of lipid-lowering therapy. Heart failure was defined as a positive response to the question ‘Do you ever have to sleep on two or more pillows to help you breathe?’ or *‘*Do you ever wake at night because you are having trouble breathing?’ and use of any of the following heart failure specific medications: angiotensin-converting enzyme inhibitor/angiotensin receptor blocker plus beta-blocker in the absence of hypertension; carvedilol; spironolactone; loop diuretics including furosemide, bumetanide, or torsemide; and/or a combination of hydralazine and nitrates.^25^

### Statistical analysis

Follow-up time was accrued from the day of the baseline in-home visit to the day the person died, the most recent follow-up date in REGARDS, or 31 December 2019, whichever came first. Baseline variables were expressed as means with standard deviations or as absolute numbers with percentages in parenthesis. Baseline biomarker values were tabulated as medians with interquartile range. Poisson regression models were used to estimate age-adjusted mortality rates per 100 person-years at risk for the total study cohort and by quartiles of biomarker levels.

We fitted Cox proportional hazard regression (HR) models with 95% confidence intervals (CIs) for all-cause and cause-specific mortality by increasing biomarker levels, adjusting for potential confounders in five sequential models: the first model was unadjusted. The second model included age, sex, race, geographic region, and an age by race interaction term (to account for the larger Black–White disparity in mortality at younger ages).^26^ The third model added cardiovascular risk factors, heart failure, and history of coronary artery disease. Model 4 added income and education, and Model 5 further included anticoagulation and antiplatelet medication use. Biomarkers were natural log transformed to approximate a normal distribution when examined as continuous variables in the Cox models. We also performed analyses by quartiles of biomarker levels, using the lowest categories as reference groups, to allow interpretable comparisons across biomarker distribution without imposing linearity assumptions. Sensitivity analysis repeated main analyses excluding participants with values of each biomarker above the 95th percentile to test the possibility that outliers due to acute illness or pre-analytic factors were impacting findings.

We tested for statistical interactions between the individual biomarkers and age, sex, race, and anticoagulation status by adding multiplicative interaction terms individually to the full Cox proportional hazards models, with *P* < 0.05 considered statistically significant. We tested the proportional hazards assumption using Schoenfeld residuals. Missing data were not imputed, and all analyses were performed as complete-case analyses. All statistical analyses were performed using Stata version 17 (Stata Corporation, College Station, TX, USA). The graph was designed using GraphPad Prism version 9 (GraphPad Software, San Diego, CA).

## Results

### Participant characteristics

A total of 2260 participants with AF and no pre-baseline stroke were included. 53.5% were women, 35.7% identified as Black, and 21.3% were taking an anticoagulant at baseline. The mean age at inclusion was 67.5 years (±9.7 years). Baseline characteristics by race are summarized in *Table 1*. White participants were slightly older, while Black participants had lower income and educational levels and were more likely to have hypertension, diabetes, and heart failure (*Table 1*). Median levels of baseline blood biomarkers were similar for the two groups, except for lower NT-proBNP and higher lipoprotein(a), IL-6, D-dimer, and factor VIII in Black participants (*Table 1*).

**Table 1:** Baseline characteristics of REGARDS participants with baseline atrial fibrillation by self-reported race

	Total (*n* = 2260)	Black (*n* = 806)	White (*n* = 1454)
Age (SD)	67 (9.7)	64 (9.2)	69 (9.6)
Male sex (%)	1050 (46.4)	276 (34.2)	774 (53.2)
Stroke Belt region (%)	1301 (57.6)	414 (51.4)	887 (61.0)
Education level ≥ high school (%)	1960 (86.8)	629 (78.2)	1331 (91.6)
Income ≥ $35 000 (%)	898 (45.7)	229 (32.6)	669 (53.1)
Smoking (ever, %)	931 (41.4)	342 (42.6)	589 (40.7)
Body mass index (kg/m^2^, SD)	29.5 ± 6.5)	31.5 ± 7.1	28.4 ± 5.8
Hypertension (%)	1525 (67.8)	653 (81.2)	872 (60.3)
Systolic blood pressure (mmHg, SD)	128 ± 17	131 ± 18	126 ± 16
History of coronary heart disease (%)	785 (35.5)	251 (32.1)	534 (37.4)
Diabetes (%)	548 (25.2)	270 (34.8)	278 (19.8)
Heart failure (%)	226 (10.0)	110 (13.7)	116 (8.0)
Dyslipidaemia (%)	1426 (65.4)	949 (67.5)	477 (61.6)
Lipid levels (mg/dL, SD)			
Total cholesterol	185 ± 41	189 ± 41	183 ± 41
LDL cholesterol	108 ± 34	113 ± 35	104 ± 33
HDL cholesterol	50 ± 17	53 ± 17	49 ± 16
Medications (%)			
Antiplatelet drugs	941 (41.6)	317 (39.3)	624 (42.9)
Warfarin^a^	482 (21.3)	84 (10.4)	398 (27.4)
Biomarkers^b^			
NT-proBNP (pg/mL)	155 (58-494)	85 (39–268)	211 (78–592)
Galectin-3 (ng/mL)	12.0 (9.4–15.0)	12.3 (9.7–15.3)	11.8 (9.2–14.8)
GDF-15 (pg/mL)	1188 (855–1729)	1130 (797–1692)	1215 (874–1743)
Cystatin C (mg/mL)	1.02 (0.87–1.24)	0.98 (0.84–1.19)	1.03 (0.89–1.27)
IL-6 (pg/mL)	3.22 (2.09–5.27)	3.42 (2.20–5.63)	3.08 (2.04–5.02)
D-dimer (µg/mL)	0.45 (0.30–0.76)	0.52 (0.33–0.89)	0.43 (0.29–0.70)
Factor VIII (%)	125 (102–154)	129 (104–162)	123 (101–151)
GGT (U/L)	23 (16–35)	17 (24–38)	22 (15–34)
Lipoprotein(a) (g/L)	0.19 (0.06–0.50)	0.40 (0.20–0.74)	0.11 (0.05–0.31)

Values are means ± standard deviations or absolute numbers with percentages in parenthesis.

^a^Direct oral coagulants were not in use at the REGARDS baseline visit.

^b^Biomarker values are medians with interquartile range in parentheses.

### Incidence of death in atrial fibrillation

During a median follow-up of 10.3 years (range 20 days to 16.8 years), 1151 persons (50.9%) died. Cardiovascular deaths accounted for 37.9% of all deaths, while 16.1% died of cancer. Other causes of death are listed in the Supplementary material (Supplementary material online, *Table S3*). The age-adjusted all-cause mortality rate was slightly lower in White participants (5.6 per 100 PY, 95% CI 5.2–6.0) than in Black participants (6.2 per 100 PY, 95% CI 5.6–6.9), but the difference was not statistically significant.

### Associations of biomarkers with mortality in atrial fibrillation

**Figure 2:**
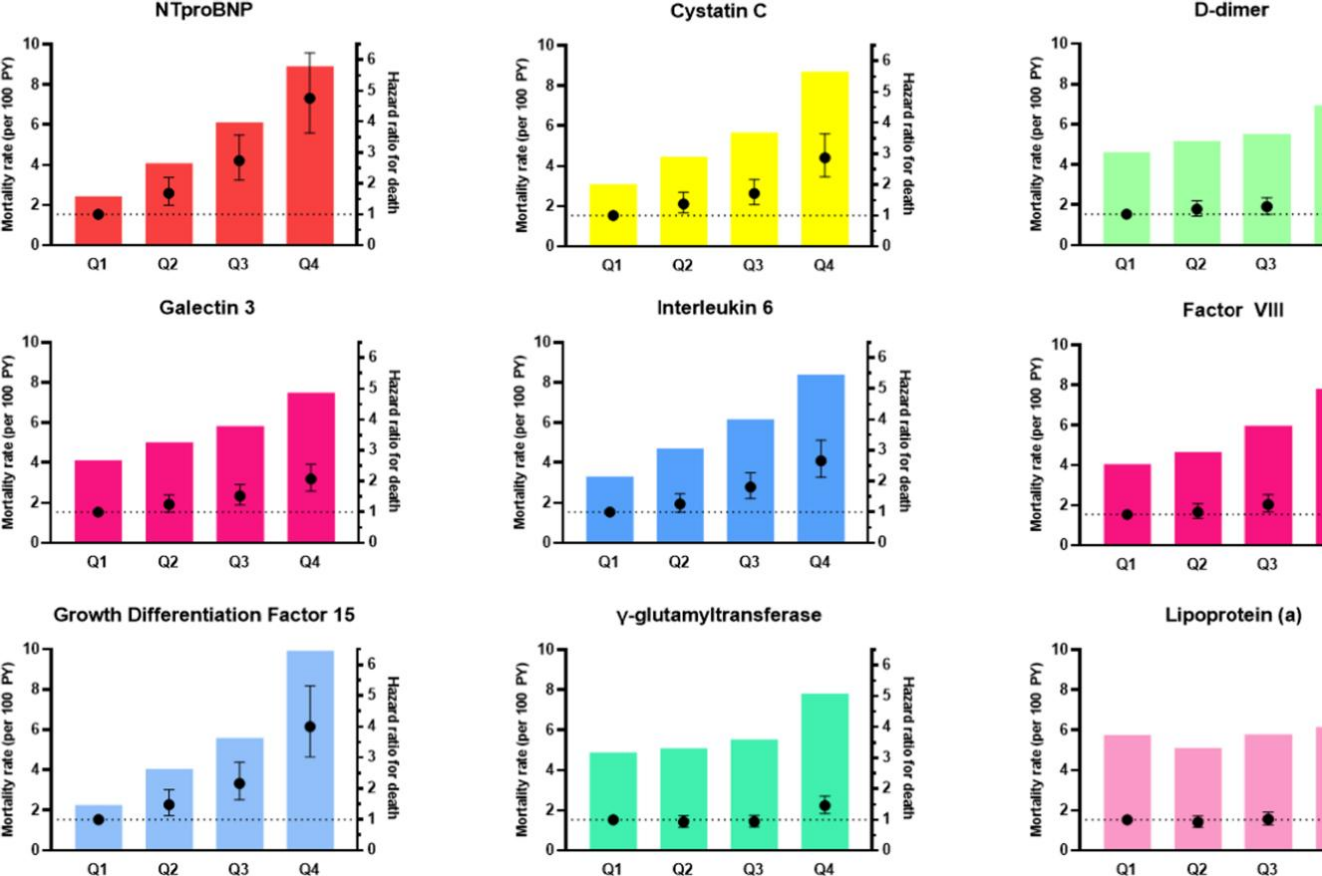
Mortality rates and hazard ratios (95% CI) for death by baseline biomarker quartiles. Mortality rates (age-adjusted) on left Y-axis (column bars), hazard ratios with 95% confidence intervals on right Y-axis (dots with range bars). Hazard ratios adjusted for age, sex, race, geographic region, body mass index, smoking (ever), hypertension, systolic blood pressure, dyslipidaemia, diabetes, heart failure, history of coronary artery disease, income status, education status, and use of anticoagulation and/or antiplatelet drugs. NT-proBNP, N-terminal pro B-type natriuretic peptide.

All-cause mortality rates across quartiles of biomarker levels are presented in *Figure 2* and Supplementary material online, *Table S4*. An apparent linear trend of increasing mortality rates by biomarker levels was observed for NT-proBNP, GDF-15, cystatin C, and IL-6. Persons with NT-proBNP or GDF-15 levels in the top quartiles had four-fold higher mortality rates compared to persons in the lowest quartiles, while nearly three-fold higher mortality rates for highest vs. lowest quartile were observed for IL-6 and cystatin C (*Figure 2*; Supplementary material online, *Table S4*). For galectin-3, D-dimer, factor VIII, and GGT, persons with biomarker levels in the highest quartile also had higher mortality rates compared to those with lower levels, but associations were more modest. We observed no difference in mortality rate across quartiles of lipoprotein(a) (*Figure 2*; Supplementary material online, *Table S4*). Excluding participants with biomarker levels above the 95th percentile did not significantly alter the mortality rate estimates (results not shown).

The individual associations of baseline biomarker levels with all-cause mortality are given in *Table 2* and *Figure 2*. In unadjusted models, increasing quartiles of biomarker levels were monotonically associated with increased risk of death for all biomarkers except GGT and lipoprotein(a). Sequential adjustment across the five models attenuated the risk estimates, but clear associations remained for most biomarkers. The greatest associations were observed for NT-proBNP and GDF-15, with HRs for death 4.85 (95% CI 3.70–6.36) and 3.98 (95% CI 3.00–5.29), respectively, for participants in the top quartile compared to the reference quartile. When examining the biomarkers as continuous variables, a 1-SD increment in log-transformed biomarker level was associated with a pronounced increased risk of death associated with NT-proBNP (HR 1.94, 95% CI 1.78–2.12), GDF-15 (HR 1.64, 95% CI 1.53–1.75), and cystatin C (1.56, 95% CI 1.46–1.68) in multivariable-adjusted analysis. A 1-SD log increase in galectin-3, IL-6, D-dimer, factor VIII, and GGT was associated with 23–31% increased risk of death (*Table 2*).

**Table 2:** Risk of death, hazard ratios with 95% confidence intervals, among people with atrial fibrillation by biomarker levels

	*N*	HR (95% confidence intervals)				
Biomarkers		Quartile 1^a^	Quartile 2	Quartile 3	Quartile 4	Per 1-SD log increase
NT-proBNP (pg/mL)		*3–57.7*	*57.8–154.6*	*154.8–492.8*	*495–35 000*	
Model 1	2120	Ref	2.13 (1.68–2.70)	4.07 (3.26–5.08)	7.59 (6.11–9.41)	
Model 2	2120	Ref	1.82 (1.43–2.32)	2.95 (2.34–3.72)	4.61 (3.65–5.81)	
Model 3	2008	Ref.	1.76 (1.37–2.27)	2.66 (2.08–3.40)	4.20 (3.29–5.38)	
Model 4	1758	Ref	1.70 (1.30–2.22)	2.66 (2.05–3.45)	4.36 (3.35–5.66)	
Model 5	1758	Ref	1.73 (1.33–2.26)	2.79 (2.15–3.63)	4.85 (3.70–6.36)	1.94 (1.78–2.12)
Galectin-3 (ng/mL)		*0.9–9.4*	*9.4–12.0*	*12.0–14.9*	*14.9–30*	
Model 1	2085	Ref	1.31 (1.08–1.59)	1.75 (1.46–2.11)	2.71 (2.27–3.24)	
Model 2	2085	Ref	1.24 (1.02–1.50)	1.58 (1.31–1.91)	2.18 (1.82–2.62)	
Model 3	1975	Ref	1.17 (0.96–1.43)	1.40 (1.15–1.71)	1.89 (1.56–2.29)	
Model 4	1729	Ref	1.24 (1.00–1.55)	1.52 (1.23–1.88)	2.04 (1.66–2.53)	
Model 5	1729	Ref	1.24 (1.00–1.55)	1.51 (1.22–1.87)	2.03 (1.65–2.51)	1.31 (1.22–1.43)
GDF-15 (pg/mL)		*194–855*	*856–1187*	*1188–1729*	*1729–6000*	
Model 1	2042	Ref	2.33 (1.82–2.98)	4.02 (3.17–5.08)	8.94 (7.12–11.2)	
Model 2	2042	Ref	1.72 (1.34–2.21)	2.45 (1.92–3.13)	5.05 (3.96–6.42)	
Model 3	1934	Ref	1.51 (1.17–1.96)	2.06 (1.60–2.66)	3.86 (2.98–5.01)	
Model 4	1931	Ref	1.49 (1.13–1.98)	2.17 (1.65–2.85)	3.99 (3.01–5.30)	
Model 5	1693	Ref	1.49 (1.13–1.97)	2.16 (1.64–2.84)	3.98 (3.00–5.29)	1.64 (1.53–1.75)
Cystatin C (mg/mL)		*0.23–0.87*	*0.88–1.02*	*1.02–1,24*	*1.24–8.19*	
Model 1	2106	Ref	1.83 (1.47–2.27)	2.86 (2.32–3.52)	5.36 (4.39–6.53)	
Model 2	2106	Ref	1.44 (1.16–1.79)	1.91 (1.55–2.36)	3.24 (2.63–3.99)	
Model 3	2000	Ref	1.35 (1.08–1.70)	1.65 (1.32–2.05)	2.67 (2.14–3.34)	
Model 4	1750	Ref	1.38 (1.08–1.76)	1.70 (1.34–2.16)	2.83 (2.23–3.60)	
Model 5	1750	Ref	1.38 (1.08–1.76)	1.69 (1.34–2.15)	2.81 (2.21–3.58)	1.56 (1.46–1.67)
IL-6 (pg/mL)		*0.34–2.09*	*2.09–3.22*	*3.22–5.27*	*4.27–1406*	
Model 1	2101	Ref	1.81 (1.48–2.21)	2.45 (2.01–2.98)	3.52 (2.91–4.26)	
Model 2	2101	Ref	1.41 (1.15–1.73)	1.99 (1.63–2.43)	2.87 (2.36–3.48)	
Model 3	1991	Ref	1.28 (1.03–1.59)	1.78 (1.44–2.20)	2.53 (2.06–3.11)	
Model 4	1744	Ref	1.25 (0.99–1.57)	1.77 (1.41–2.22)	2.62 (2.10–3.27)	
Model 5	1744	Ref	1.25 (0.99–1.57)	1.76 (1.40–2.21)	2.61 (2.08–3.26)	1.29 (1.22–1.37)
D-dimer (µg/mL)		*0.03–0.30*	*0.30–0.45*	*0.46–0.76*	*0.77–20*	
Model 1	2069	Ref	1.19 (0.98–1.44)	1.54 (1.29–1.85)	2.23 (1.88–2.66)	
Model 2	2069	Ref	1.13 (0.93–1.37)	1.26 (1.05–1.51)	1.66 (1.39–2.00)	
Model 3	1959	Ref	1.15 (0.94–1.39)	1.19 (0.98–1.43)	1.55 (1.29–1.88)	
Model 4	1712	Ref	1.09 (0.88–1.35)	1.13 (0.92–1.39)	1.58 (1.29–1.94)	
Model 5	1712	Ref	1.16 (0.93–1.43)	1.22 (0.98–1.51)	1.74 (1.40–2.15)	1.29 (1.20–1.39)
Factor VIII (%)		*13–102*	*102–125*	*125–154*	*154–800*	
Model 1	2080	Ref	1.31 (1.08–1.60)	1.90 (1.57–2.89)	2.65 (2.21–3.18)	
Model 2	2080	Ref	1.15 (0.94–1.40)	1.48 (1.23–1.79)	2.17 (1.81–2.62)	
Model 3	1970	Ref	1.10 (0.90–1.35)	1.39 (1.15–1.69)	1.98 (1.63–2.40)	
Model 4	1721	Ref	1.08 (0.87–1.35)	1.31 (1.07–1.62)	2.05 (1.67–2.53)	
Model 5	1721	Ref	1.08 (0.87–1.35)	1.32 (1.07–1.62)	2.03 (1.65–2.50)	1.31 (1.22–1.41)
GGT (U/L)		*3–16*	*16–23*	*23–35*	*36–655*	
Model 1	2081	Ref	0.94 (0.79–1.12)	1.05 (0.89–1.25)	1.45 (1.23–1.71)	
Model 2	2081	Ref	0.98 (0.82–1.17)	1.07 (0.90–1.28)	1.59 (1.35–1.88)	
Model 3	1971	Ref	0.98 (0.82–1.17)	1.00 (0.83–1.19)	1.45 (1.22–1.73)	
Model 4	1725	Ref	0.93 (0.77–1.13)	0.97 (0.79–1.18)	1.48 (1.23–1.78)	
Model 5	1725	Ref	0.93 (0.76–1.13)	0.96 (0.79–1.17)	1.46 (1.21–1.76)	1.23 (1.14–1.32)
Lipoprotein(a) (g/L)		*0.026–0.064*	*0.065–0.189*	*0.19–0.501*	*0.504–2.81*	
Model 1	2081	Ref	0.91 (0.77–1.08)	0.97 (0.82–1.15	0.99 (0.84–1.18)	
Model 2	2081	Ref	0.86 (0.72–1.02)	0.96 (0.81–1.15)	1.07 (0.89–1.29)	
Model 3	1971	Ref	0.91 (0.76–1.08)	0.96 (0.80–1.16)	1.10 (0.91–1.33)	
Model 4	1725	Ref	0.90 (0.74–1.09)	1.00 (0.82–1.22)	1.09 (0.89–1.34)	
Model 5	1725	Ref	0.90 (0.74–1.10)	1.01 (0.83–1.23)	1.09 (0.89–1.34)	1.03 (0.96–1.12)

Model 1: unadjusted model; Model 2: adjusted for age, sex, race, geographic region, age*race interaction; Model 3: adjusted for Model 3 variables, body mass index, smoking (ever), hypertension, systolic blood pressure, dyslipidaemia, diabetes, heart failure, and history of coronary artery disease; Model 4: adjusted for all Model 3 variables, income, and education level; Model 5: adjusted for all Model 4 variables, use of antiplatelet drugs, and use of anticoagulation.

^a^Interquartile range in *italic*.

Evaluating the proportional hazards assumption using Schoenfeld residuals revealed a potential violation of the proportional hazards assumption for NT-proBNP, galectin-3, and D-dimer, suggesting that the association of these biomarkers with mortality is not constant over time. To address this, we examined the risk of death by these biomarkers in time intervals after study start. For all three biomarkers, the association was most pronounced in the first 5 years after study start and weakened during follow-up, although the association after 10 years of follow-up remained increased for all three biomarkers and was robust for NT-proBNP [HR per 1SD increment 2.08 (1.79–2.40) in the first 5 years and 1.76 (1.50–2.07) after 10 years; Supplementary material online, *Table S5*].

### Interactions of biomarkers with age, race, or anticoagulant use

For most of the examined biomarkers, we did not identify significant interactions with age, race, or use of anticoagulation. Mortality risk estimates were more pronounced for men than women for galectin-3, factor VIII, D-dimer, and GGT (all *P* for interaction < 0.05). While there was no association for lipoprotein(a) and all-cause mortality in the full cohort, a 1-SD higher log[lipoprotein(a)] was associated with a 26% increased risk of death in persons under 65 years of age (HR 1.26; 95% CI 1.05–1.48), while the HR was 1.05: 0.98–1.13 in those ≥65 (*P* for interaction 0.005). The only interaction by race was observed for factor VIII; the HR was higher for White persons than for Black persons [HR per SD increment 1.38 (95% CI 1.25–1.53) vs. 1.24 (95% 1.11–1.39); *P* for interaction 0.04]. Participants using warfarin at baseline had weaker associations of D-dimer with mortality than those not using anticoagulation [HR per SD increment 1.13 (95% CI 0.98–1.30) vs. 1.37 (95% CI 1.26–1.49); *P* for interaction 0.04] (see Supplementary material online, *Table S6*).

### Associations of biomarkers with different causes of mortality

In analyses of cause-specific mortality (*Table 3*), we observed an increased risk of cardiovascular death by higher biomarker levels for all included biomarkers, with the most pronounced associations for NT-proBNP, GDF-15, and cystatin C (*Table 3*). A 1-SD log(NT-proBNP) increase was associated with a more than two-fold increased risk of cardiovascular death (HR 2.17, 95% CI 1.89–2.49), and those with NT-proBNP values in the upper quartile had a seven-fold increased risk of cardiovascular death compared to persons in the first quartile (HR 7.19, 95% CI 4.35–11.9) (*Table 3*). NT-proBNP, GDF-15, IL-6, GGT, and D-dimer were associated with cancer death, with HRs ranging from 1.19 (95% CI 1.02–1.39) per 1-SD increment in log(IL-6) to 1.36 (95% CI 1.08–1.69) per 1-SD increment in log(NT-proBNP).

**Table 3:** Risk of cardiovascular and cancer death, hazard ratios with 95% confidence intervals, among people with atrial fibrillation by biomarker levels^a^

Biomarkers	Adjusted HR (95% CI)				
	Quartile 1	Quartile 2	Quartile 3	Quartile 4	Per 1 SD log increase
NT-proBNP					
CVD death	Ref	2.43 (1.47–4.03)	4.06 (2.48–6.65)	7.19 (4.35–11.9)	2.17 (1.89–2.49)
Cancer death	Ref	0.91 (0.52–1.57)	1.52 (0.89–2.60)	1.93 (1.07–3.46)	1.36 (1.08–1.69)
Galectin-3					
CVD death	Ref	1.38 (0.90–1.98)	1.52 (1.06–2.17)	2.10 (1.48–2.97)	1.33 (1.17–1.51)
Cancer death	Ref	1.31 (0.77–2.21)	1.74 (1.04–2.87)	1.68 (0.98–2.88)	1.14 (0.94–1.38)
GDF-15					
CVD death	Ref	0.97 (0.62–1.50)	1.44 (0.94–2.20)	2.93 (1.90–4.51)	1.60 (1.43–1.78)
Cancer death	Ref	1.60 (0.85–3.01)	2.92 (1.58–5.39)	2.12 (1.05–4.29)	1.21 (1.01–1.45)
Cystatin C					
CVD death	Ref	0.98 (0.67–1.43)	1.17 (0.81–1.69)	2.03 (1.41–2.93)	1.48 (1.33–1.66)
Cancer death	Ref	1.17 (0.70–1.97)	1.29 (0.76–2.18)	1.37 (0.77–2.41)	1.16 (0.94–1.43)
Interleukin-6					
CVD death	Ref	1.17 (0.77–1.63)	1.55 (1.07–2.24)	2.53 (1.78–3.62)	1.27 (1.16–1.38)
Cancer death	Ref	0.98 (0.57–1.69)	1.80 (1.08–3.08)	1.75 (1.02–2.99)	1.19 (1.02–1.39)
GGT					
CVD death	Ref	1.04 (0.76–1.43)	0.87 (0.63–1.22)	1.36 (0.99–1.85)	1.16 (1.03–1.30)
Cancer death	Ref	0.93 (0.57–1.53)	1.17 (0.72–1.91)	1.58 (0.96–2.59)	1.21 (1.01–1.48)
D-dimer					
CVD death	Ref	1.29 (0.91–1.83)	1.24 (0.87–1.78)	1.98 (1.40–2.80)	1.35 (1.20–1.51)
Cancer death	Ref	1.08 (0.64–1.83)	1.27 (0.75–2.11)	1.37 (0.79–2.37)	1.27 (1.05–1.53)
Factor VIII					
CVD death	Ref	1.02 (0.71–1.45)	1.23 (0.88–1.73)	1.88 (1.35–2.63)	1.26 (1.12–1.52)
Cancer death	Ref	0.76 (0.46–1.28)	0.86 (0.52–1.42)	1.39 (0.85–2.26)	1.20 (0.99–1.45)
Lipoprotein(a)					
CVD death	Ref	1.19 (0.85–1.67)	1.35 (0.96–1.89)	1.44 (1.02–2.04)	1.14 (1.01–1.29)
Cancer death	Ref	0.58 (0.35–0.96)	0.71 (0.43–1.17)	0.90 (0.55–1.15)	0.98 (0.81–1.19)

^a^Per 1-SD increment in log(biomarker) value, adjusted for age, sex, race, geographic region, age*race interaction, body mass index, smoking (ever), hypertension, systolic blood pressure, dyslipidaemia, diabetes, heart failure, history of coronary artery disease, income, education level, use of platelet drugs, and use of anticoagulation.

## Discussion

In this large population-based cohort study of individuals with AF, we observed consistent and monotonically increased mortality risk for biomarkers of cardiovascular stress and dysfunction (NT-proBNP, GDF-15), myocardial fibrosis (galectin-3), renal dysfunction (cystatin C), inflammation (IL-6), and coagulation activity (D-dimer and factor VIII). Associations were more prominent for cardiovascular than cancer mortality. Only for factor VIII was there a significant difference by race, where factor VIII was associated with a slightly higher all-cause mortality risk in White participants. The association of D-dimer with mortality was weaker in those using vs. not using anticoagulants at baseline.

Our findings that markers of cardiac stress, inflammation, and coagulation are risk factors for all-cause and cardiovascular mortality are in line with findings from large clinical trials for anticoagulant treatment in AF.^7,9^ In both the RE-LY and ARISTOTLE trials, sub-studies identified consistent and strong association with mortality for NT-proBNP, GDF-15, cystatin C, IL-6, factor VIII, and D-dimer.^8,9,20,27,28^ A recent cohort study from a tertiary care setting in Austria similarly found associations of higher NT-proBNP, D-dimer, and GDF-15 with mortality in AF.^29^ Our study adds to these findings by confirming associations in a substantially larger, real-world cohort including both Black and White participants and with long-term follow-up. We found stronger risk estimates for cardiovascular mortality than cancer mortality by individual biomarker levels, with the most pronounced difference observed for NT-proBNP. In agreement with our findings, the RE-LY trial reported an almost seven-fold increased risk for vascular death for persons in the highest vs. the lowest tertile of NT-proBNP but, unlike our findings, found no significant association between NT-proBNP and non-vascular death.^28^ The stronger associations of most biomarkers with cardiovascular than cancer mortality likely reflect their greater relevance to pathways driving cardiovascular disease rather than oncogenesis or cancer progression.

GGT has been implicated as a risk factor for all-cause mortality,^30^ fatal cardiovascular disease,^31^ and AF development,^32^ but we are not aware of other studies on the impact of GGT on mortality in AF. The excess risk of death in those in the highest GGT quartile may reflect concurrent liver disease in these participants, as concomitant AF has previously been shown to increase mortality risk in patients with hepatic cirrhosis.^33,34^ Also, coexisting liver disease may complicate anticoagulation treatment, leading to higher rates of adverse outcomes. While Lp(a) has not previously been investigated as a risk factor for AF mortality, a recent meta-analysis reported significant associations for Lp(a) and cardiovascular mortality in both the general population and in individuals with chronic diseases but, in agreement with our findings, found no association for non-CVD death.^35^

In the present analysis, associations of GGT, galectin-3, factor VIII, and D-dimer with mortality were greater in men than women, and the association of factor VIII was greater in White than Black participants. To our knowledge, this is the first study to report on sex and race differences in mortality risk by these biomarkers in AF. Studies on sex-specific associations and adverse outcomes in non-AF populations have yielded diverging results. In the population-based ARIC cohort, no interaction was found between galectin-3 levels and sex for mortality,^36^ whereas increasing levels were more strongly associated with heart failure in women than men in large pooled study from four community-based cohorts.^37^ Another ARIC report found increased risk of AF development by factor VIII, with similar relative risks for men and women. In this ARIC study, increasing factor VIII levels were also associated with excess mortality in both AF and non-AF participants, but these analyses were not stratified on sex.^38^ The racial difference in the association of factor VIII with mortality in the present study is in contrast to previous REGARDS findings of similar associations of factor VIII with both stroke and coronary heart disease by race.^39^ We only observed an interaction by anticoagulation use for D-dimer, where the risk of death with higher D-dimer was greater in non-users than in users. Previous studies have demonstrated that higher D-dimer levels while on anticoagulant therapy is associated with adverse outcomes, including for stroke in AF in REGARDS.^40^ Anticoagulation also reduces D-dimer levels.^41^ Since D-dimer is a procoagulant marker, our findings may suggest that anticoagulant therapy could be beneficial in persons with higher D-dimer, but we can only speculate on this at this time. Further studies are needed to confirm our findings, elucidate underlying mechanisms, and assess possible interventions.

The main strengths of our study include the large number of both Black and White participants recruited from a geographically diverse population; the prospective, population-based design; long-term follow-up; multiple biomarkers measured; and the carefully validated outcomes. Previous data on these biomarkers in AF mortality were mostly derived from anticoagulated trial populations and thus not necessarily generalizable to other populations. We were able to investigate the impact of age, sex, and race on mortality and stratify subgroup analyses by these variables, thus expanding on previous work. Several limitations should also be considered. The AF exposure was not adjudicated for all participants, as it was defined by either electrocardiogram or a self-reported history of AF. While either definition was similarly predictive of stroke in REGARDS,^22^ misclassification bias cannot be ruled out. The REGARDS study was not primarily designed to study AF outcomes, and information on changes in participants’ medication use, including anticoagulation, was not collected frequently. Also, temporal changes in the surveillance and treatment of AF over time may bias effect estimates. We did not measure the group of biomarkers in REGARDS participants without AF, so we cannot say the observed associations are unique to AF. But, since those with AF have higher mortality than the general population, findings remain important to understanding. Biomarkers were measured at baseline only and may have fluctuated over time. Single time point measurement of any modifiable risk factor is a potential limitation in clinical research, especially when follow-up is long; the resulting non-differential misclassification generally leads to regression dilution bias, underestimating the true associations between exposure and outcome. Biomarker stability may also be an issue when analysing plasma samples stored over time, considering the potential effects of sample handling, freeze-thaw cycles, and transportation conditions on sample quality. A prior study from our laboratory found no consistent storage time effect upon assayed values of multiple risk biomarkers for atherosclerosis and thrombosis.^42^ We corrected for assay drift when applicable using previously established methods (see Supplementary material online, *Table S2*).^42^ Despite multivariable adjustments and stratified analyses, we cannot completely exclude residual confounding that may have impacted our results.

The clinical implementation of using the studied biomarkers in AF risk prediction remains an ongoing research area, primarily focused on improving anticoagulant treatment decisions for stroke prevention. In a sub-study of the RE-LY and ARISTOTLE trials, the addition of NT-proBNP, GDF-15, and troponin T to the ABC (age, biomarkers, clinical history) death score significantly improved risk prediction of both all-cause and cardiovascular death in individuals with AF compared to clinical variables alone.^8^ In REGARDS, addition of NT-proBNP and GDF-15 to the CHA_2_DS_2_-VASc score improved stroke risk prediction in those not taking anticoagulation at baseline.^43^ Our confirmation of the biomarker–mortality relationship in a biracial cohort adds to these findings. Implementing biomarkers in AF risk stratification may aid in clinical decision-making, such as identifying patients who require intensified cardiovascular risk management or more aggressive rhythm-control strategies.

In conclusion, several widely available biomarkers were associated with both all-cause and cardiovascular mortality in AF. Our results suggest that integrating biomarker assessment in persons with AF may lead to more targeted interventions to reduce mortality.

## Supplementary Material

oeag022_Supplementary_Data

## Data Availability

In cooperation with the Institutional Review Board of the University of Alabama at Birmingham, the REGARDS project facilitates data sharing through formal data use agreements. Investigators who wish to access the data should send their requests to regardsadmin@uab.edu.
