# Supplementary material for: Circulating biomarkers and mortality in atrial fibrillation: the REasons for Geographic And Racial Differences in Stroke study
Source: Eur Heart J Open. 2026 Feb 17;6(2):oeag022. doi: 10.1093/ehjopen/oeag022 (PMC13043327; doi:10.1093/ehjopen/oeag022)
Supplement: oeag022_Supplementary_Data [file oeag022_supplementary_data.docx]

**SUPPLEMENTAL MATERIALS**

**Circulating Biomarkers and Mortality in Atrial Fibrillation: The Reasons for**

**Geographic And Racial Differences in Stroke Study**

Erin M. Hald, MD, PhD^1,2^, Katherine Wilkinson, MS^1^, Samuel A. P. Short, MD^1^, Suzanne E. Judd, PhD^3^, Virginia J. Howard, PhD^4^, Emily B. Levitan, ScD^4^, Elsayed Z. Soliman, MD, MSc^5^, Mary Cushman, MD, MSc^1^

^1^Department of Medicine, Larner College of Medicine at the University of Vermont, Burlington, VT, USA

^2^Thrombosis Research and Expertise Center (TREC), Department of Clinical Medicine, UiT-The Arctic University of Norway, Tromsø, Norway

^3^Department of Biostatistics, University of Alabama at Birmingham, Birmingham, Alabama, USA.
^4^Department of Epidemiology, University of Alabama at Birmingham, Birmingham, AL, USA
^5^Epidemiological Cardiology Research Center, Department of Internal Medicine, Section on Cardiovascular Medicine, Wake Forest School of Medicine, Winston-Salem, NC, USA.


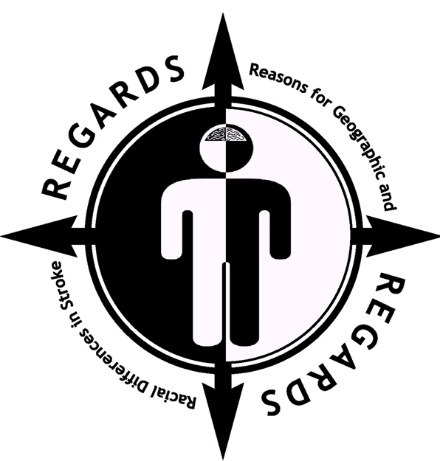


**TABLE OF CONTENTS**

**Supplemental Methods 3**

Laboratory methods and data harmonization 3

Blood collection, storage, and biomarker assays 3

Supplemental Table 1: Biomarker assay characteristics 4

Supplemental Table 2: Biomarker assay harmonization steps 6

**Data Availability, Reproducibility, and Rigor 7**

**Supplemental Data Tables**

Supplemental Table 3. Adjudicated causes of death for the study participants 8

Supplemental Table 4. Age-adjusted all-cause mortality rates per 100 person-

years (95% CI) 9

Supplemental Table 5. Hazard ratios for al-cause mortality with 95%

confidence intervals by time periods after study start 10

Supplemental Table 6. Hazard ratios for mortality per 1 SD increment in

log(biomarker)with 95% confidence intervals by age groups, sex, race and

anticoagulation use 11

**Supplemental Methods**

**Laboratory methods and data harmonization**

Blood collection, storage, and biomarker assays

Blood samples were obtained using standardized methods, centrifuged within 120 minutes of sampling, and shipped overnight on ice to a central laboratory and biorepository, the University of Vermont Laboratory for Clinical Biochemistry Research (LCBR).^23^ Samples were stored at -80C until batch analysis. All assays were performed with commercially available kits by trained technicians (Supplemental Table 1). As multiple biomarkers studied here were measured previously in some of these participants for other studies dating back to 2011, we adopted a conservative assay approach to steward repository resources and reduce costs. Specifically, we utilized previously measured results when available and corrected for assay drift when applicable using methods in place in the LCBR for decades (Supplemental Table 2).^42^

**Supplemental Table 1: Biomarker assay characteristics**

| **Measurement** | **Parameters** | **Biomarker** | | | | | | | |
| --- | --- | --- | --- | --- | --- | --- | --- | --- | --- |
|  |  | **D-dimer** | **Factor VIII antigen** | **Galectin 3** | **GDF-15** | **GGT** | **IL-6** | **Lp(a)** | **NTproBNP** |
| 2022 AF Run | Assay | Stago STA-R Evolution | Affinity Biologicals (ERL) ELISA | R&D Elisa | R&D Elisa | Roche E411 | MSD  ECLIA | Siemans BNII | Roche E411 |
|  | Interassay CV | 1.03-25.3%* | 5.4%-6.7% | 7.73-10.16% | 6.73-7.10% | 0.7-5.4% | 7.2-9.3% | 5.7-8.4% | 2.9-8.7% |
| 2021 BioMedioR Run | Assay | Stago STA-R Evolution | ERL ELISA EIA | - | - | - | MSD  ECLIA | - | Roche E411 |
|  | Interassay CV | 5 -14% | 5 - 8% | - | - | - | 3.94-6.87% | - | <5% |
| 2011 Stroke Case/Random Sample Run | Assay | Diagnostica Stago STA-R | ERL  ELISA | R&D ELISA | - | - | R&D HS ELISA | Siemans BNII | - |
|  | Interassay CV | 5-14% | 15% | 5.57-7.39% | - | - | 6.3% | 2.0–5.3%. | - |
|  |  | - | - | - | - |  | - | - | - |
| 2013 GGT Run in Stroke Case/Random Sample Cohort | Assay | - | - | - | - | Roche Elecsys | - | - | - |
|  | Interassay CV | - | - | - | - | 0.74-2.95% | - | - |  |
| 2015 NTproBNP Run | Assay | - | - | - | - | - | - | - | Roche Elecsys |
|  | Interassay CV | - | - | - | - | - | - | - | <5% |

*High CV due to low mean values. Commercial assays were obtained from Affinity Biologicals (Ontario, Canada), Diagnostica Stago (Asnières-sur-Seine, France), Enzyme Research Lab (ERL; Indiana, USA), MesoScale Diagnosics (MSD; Maryland, USA), R&D Systems (Minnesota,SA), Roche (Basel, Switzerland), and Siemans (Erlangen, Germany). The REGARDS stroke/random sample and BioMedioR, (biomarkers as mediators of racial disparities in risk factors nested subcohort) were previously described.^44,45^

**Supplemental Table 2: Biomarker assay harmonization steps**

| **Biomarker** | **Harmonization considerations** | | |
| --- | --- | --- | --- |
|  | **Assay differences** | **Assay drift by embedded lyophilized control samples** | **Assay drift by**  **Participant sample re-runs** |
| **D-dimer** | None | No drift | No drift |
| **Factor VIII antigen** | None | No drift | Data from 2011 (Stroke C/RS) were 9 ag% higher, compared to later measures (no drift between BioMedioR and AF visits). 9 ag% were added to measures from BioMedioR and the AF runs. |
| **Galectin-3** | None | No drift | No drift |
| **GDF-15** | NA (single measurement) | NA (single measurement) | NA (single measurement) |
| **GGT** | None | No drift | No drift |
| **IL-6** | MSD and R&D results were not co-linear. All measurements by MSD were divided by 0.3868 to compare directly to R&D. | After transformation for assay differences, there was no drift between measures in 2011 (Stroke C/RS) and 2021 (BioMedioR).  2022 values (AF visits 1 and 2) were 23.7% higher than 2021 (BioMedioR). All values from 2022 were divided by a factor of 1.237. | After correcting for assay differences and drift by control set, results from 2011 (stroke C/RS) were 0.16 pg/mL higher than later measures (2021-22 BioMedioR and AF visit 1, no drift between these measures). All results later than 2011 were raised by 0.16 pg/mL. |
| **Lp(a)** | None | No drift | None |
| **NTproBNP** | None | No drift | No drift |

Stroke C/RS, REGARDS stroke case and random sample sub-cohort^44^ BioMedioR, REGARDS Biomarkers and MEDiatoris of racial disparities in Risk factors nested subcohort.^45^

**Data Availability, Reproducibility, and Rigor**

REGARDS data are not publicly available due to ethical and legal restrictions. To abide by its obligations with NIH/NINDS and the Institutional Review Board of the University of Alabama at Birmingham, REGARDS facilitates data sharing through data use

agreements. Any investigator is welcome to access the REGARDS data, including statistical code, through this process. Requests for data access may be sent to [regardsadmin@uab.edu](mailto:regardsadmin@uab.edu). According to REGARDS policy, the aims and analysis plan for this

manuscript were prespecified and reviewed and approved by the REGARDS publications committee, which also reviewed the final manuscript and assured the *a priori* plans were followed.

**Supplemental Table 3. Adjudicated causes of death for the study participants**

| **Biomarkers** | **N (%)** | |
| --- | --- | --- |
| Cardiovascular disease | | 436 (37.9) |
| Cancer | | 185 (16.1) |
| Accidents/injury/suicide | | 36 (3.1) |
| Liver disease | | 7 (0.6) |
| Infectious diseases | | 142 (12.3) |
| End-stage renal disease | | 31 (2.7) |
| Dementia | | 86 (7.5) |
| Respiratory disease | | 76 (6.6) |
| Pulmonary embolism | | 9 (0.8) |
| Other non-cardiac, non-stroke death | | 42 (4.5) |
| Unclassifiable | | 91 (7.9) |

**Supplemental Table 4. Age-adjusted all-cause mortality rates per 100 person-years (95% CI)**

| **Biomarkers** | **N** | **Quartile 1** | **Quartile 2** | **Quartile 3** | **Quartile 4** |
| --- | --- | --- | --- | --- | --- |
| NT-proBNP | 2120 | 2.43 (1.94-2.92) | 4.09 (3.51-4.67) | 6.11 (5.45-6.77) | 8.93 (8.06-9.79) |
| Galectin-3 | 2085 | 4.11 (3.50-4.71) | 5.00 (4.34-5.65) | 5.84 (5.15-6.52) | 7.50 (6.72-8.28) |
| GDF-15 | 2042 | 2.26 (1.78-2.75) | 4.04 (3.47-4.60) | 5.59 (4.95-6.22) | 9.93 (8.96-10.9) |
| Cystatin C | 2106 | 3.11 (2.55-3.67) | 4.47 (3.88-5.06) | 5.68 (5.04-6.32) | 8.71 (7.85-9.57) |
| IL-6 | 2101 | 3.31 (2.77-3.85) | 4.69 (4.11-5.28) | 6.18 (5.48-6.89) | 8.39 (7.53-9.26) |
| D-dimer | 2069 | 4.61 (3.96-5.25) | 5.18 (4.49-5.88) | 5.52 (4.87-6.17) | 6.96 (6.21-7.70) |
| FVIII | 2080 | 4.04 (3.44-4.65) | 4.65 (4.04-5.26) | 5.99 (5.30-6.67) | 7.81 (6.99-8-63) |
| GGT | 2081 | 4.89 (4.32-5.46) | 5.10 (4.44-5.75) | 5.54 (4.83-6.25) | 7.82 (6.92-8.71) |
| Lp(a) | 2081 | 5.75 (5.06-6.43) | 5.12 (4.49-5.75) | 5.79 (5.08-6.50) | 6.15 (5.40-6.90) |

**Supplemental Table 5. Hazard ratios for all-cause mortality* with 95% confidence intervals by time periods after study start**

|  | NT-proBNP | Galectin-3 | D-dimer |
| --- | --- | --- | --- |
| Time after study start |  |  |  |
| 0-5 years | 2.08 (1.79-2.40) | 1.47 (1.27-1.69) | 1.45 (1.28-1.64) |
| 5-10 years | 1.86 (1.60-2.16) | 1.31 (1.15-1.50) | 1.25 (1.10-1.42) |
| 10 y to study end | 1.76 (1.50-2.07) | 1.18 (1.03-1.36) | 1.15 (1.00-1.32) |

*per 1 SD increment in log(biomarker) value, adjusted for age, sex, race, geographic region, body mass index, smoking (ever), hypertension, systolic blood pressure, dyslipidemia, diabetes, heart failure, history of coronary artery disease, income, use of platelet drugs and use of anticoagulation

**Supplemental Table 6. Hazard ratios (HR) for all-cause mortality with 95% confidence intervals (CI) per 1 SD increment in log(biomarker), stratified by age groups, sex, race and anticoagulation use***

*All shown stratified analyses - p for interaction <0.05

†Analyses are adjusted for age, sex, race, age*race interaction, geographic region, body mass index, smoking (ever), hypertension, systolic blood pressure, dyslipidemia, diabetes, heart failure, history of coronary artery disease, income, use of platelet drugs and use of anticoagulation. The stratifying factor (e.g. age) is removed from the model stratifying on that variable (e.g. Age group).

|  | **Age group†** | | **Sex** | | **Race** | | **Anticoagulation use** | |
| --- | --- | --- | --- | --- | --- | --- | --- | --- |
| **Biomarker** | **<65** | **≥ 65** | **Female** | **Male** | **White** | **Black** | **No** | **Yes** |
| Galectin-3 |  |  | 1.15 (1.01-1.28) | 1.46 (1.31-1.62) |  |  |  |  |
| Factor VIII | 1.51 (1.31-1.75) | 1.31 (1.21-1.42) | 1.22 (1.10-1.34) | 1.45 (1.29-1.62) | 1.38 (1.25-1.53) | 1.24 (1.11-1.39) |  |  |
| D-dimer |  |  | 1.18 (1.05-1.32) | 1.39 (1.26-1.54) |  |  | 1.37 (1.26-1.39) | 1.13 (0.98-1.30) |
| GGT |  |  | 1.11 (0.99-1.23) | 1.38 (1.25-1.53) |  |  |  |  |
| Lipoprotein (a) | 1.26 (1.07-1.48) | 1.05 (0.98-1.13) |  |  |  |  |  |  |
